# The association of genomic lesions and PD-1/PD-L1 expression in resected triple-negative breast cancers

**DOI:** 10.1186/s13058-018-1004-0

**Published:** 2018-07-11

**Authors:** Michael T. Barrett, Elizabeth Lenkiewicz, Smriti Malasi, Anamika Basu, Jennifer Holmes Yearley, Lakshmanan Annamalai, Ann E. McCullough, Heidi E. Kosiorek, Pooja Narang, Melissa A. Wilson Sayres, Meixuan Chen, Karen S. Anderson, Barbara A. Pockaj

**Affiliations:** 1 Division of Hematology and Medical Oncology, Mayo Clinic in Arizona, Scottsdale, AZ USA; 2 Merck Research Laboratories, Palo Alto, CA USA; 3 Department of Pathology and Laboratory Medicine, Mayo Clinic in Arizona, Scottsdale, AZ USA; 4 School of Life Sciences, Arizona State University, Tempe, AZ USA; 5 Biodesign Institute, Arizona State University, Tempe, AZ USA; 6 Division of Hematology and Medical Oncology, Mayo Clinic in Arizona, Phoenix, AZ USA; 7 Division of General Surgery, Section of Surgical Oncology, Mayo Clinic in Arizona, Phoenix, AZ USA

**Keywords:** PD-1, PD-L1, IHC, Flow sorting, Copy number, Somatic mutations, Triple-negative breast cancer

## Abstract

**Background:**

Elevated PD-L1 expression on tumor cells, a context associated with an adaptive immune response, has been linked to the total burden of copy number variants (CNVs) in aneuploid tumors, to microsatellite instability (MSI), and to specific genomic driver lesions, including loss of *PTEN*, *MYC* amplification, and activating mutations in driver oncogenes such as *KRAS* and *PIK3CA*. Triple-negative breast cancers (TNBCs) typically have high levels of CNVs and diverse driver lesions in their genomes. Thus, there is significant interest in exploiting genomic data to develop predictive immunotherapy biomarkers for patients with TNBC.

**Methods:**

Whole tissue samples from 55 resected TNBCs were screened by immunohistochemistry (IHC) for PD-1 and PD-L1 by using validated antibodies and established scoring methods for staining of tumor and non-tumor cells. In parallel, we interrogated biopsies from each resection with DNA content flow cytometry and sorted the nuclei of diploid, tetraploid, and aneuploid cell populations. CNVs were mapped with CNV oligonucleotide arrays by using purified (>95%) tumor populations. We generated whole exome data for 12 sorted tumor samples to increase the resolution within loci of interest and to incorporate somatic mutations into our genomic signatures.

**Results and Conclusions:**

PD-L1 staining was detected on tumor cells in 29 out of 54 (54%) evaluable cases and was associated with increased overall survival (*P* = 0.0024). High levels of PD-1 and PD-L1 (IHC ≥4) were present in 11 out of 54 (20%) and 20 out of 54 (37%) cases with staining of PD-L1 primarily on tumor cells for 17 out of 20 (85%) cases. The latter included tumors with both high (>50) and low (<20) numbers of CNVs. Notably, homozygous deletion of *PTEN* (*n* = 6) or activating mutation in *PIK3CA* (*n* = 1) was not associated with increased expression of either immune checkpoint activator in TNBC. In contrast, two treatment-naïve cases with *EGFR* driver amplicons had high PD-L1 tumor staining. High mutational load and predicted neoepitopes were observed in MSI^+^ and high CNV burden TNBCs but were not associated with high PD-L1 expression on tumor cells. Our results challenge current models of genomic-based immunotherapy signatures yet suggest that discrete genomic lesions may complement existing biomarkers to advance immune checkpoint therapies for patients with TNBC.

**Electronic supplementary material:**

The online version of this article (10.1186/s13058-018-1004-0) contains supplementary material, which is available to authorized users.

## Background

Multiple studies suggest that high levels of PD-L1 on tumor cell surfaces are associated with an adaptive immune resistance in the presence of active tumor-infiltrating lymphocytes (TILs) [1, 2]. Thus, this immunohistochemistry (IHC) staining pattern represents a candidate signature for those tumors that can be effectively targeted with checkpoint blockade. An emerging picture suggests that tumor-specific genomic lesions, either individually or in combination, are associated with immune checkpoint activation and the extent and duration of responses for patients to immunotherapy. These lesions include loss of tumor suppressor genes (*PTEN*), the activation of oncogenic drivers (*EGFR*, *KRAS*, and *PIK3CA*), BRCA mutant and BRCA-like homologous recombination-deficient (HRD) genomes, and high mutation burdens, including microsatellite instability (MSI), chromosomal instability (CIN), and aneuploidy [3–9]. The highly aberrant nature of triple-negative breast cancer (TNBC) genomes makes TNBC a highly favorable model to test genomic correlates of PD-1 and PD-L1 expression [10].

In this study, we interrogated a series of 55 well-annotated surgical resections from patients with TNBC with IHC for PD-1 and PD-L1 protein expression by using validated antibodies and established scoring methods that included PD-L1 staining intensities on tumor and non-tumor cells [11]. The expression patterns were correlated with clinical outcomes. We then assessed the associations of genomic lesions with expression of PD-1 and PD-L1 in each sample. We applied a systematic approach to rigorously interrogate the genomes of each TNBC sample. Tumor ploidy was initially measured with DNA content flow cytometry followed by sorting of the nuclei of distinct diploid, tetraploid, and aneuploid cell populations from each TNBC. Thus, rather than inferring ploidy on the basis of sequencing reads or single-nucleotide polymorphism (SNP) arrays, we used the direct measure of total DNA from our flow assays. The next level of analysis incorporated genome-wide copy number variant (CNV) measures with oligonucleotide arrays designed for CNV detection using purified (>95%) flow-sorted tumor populations. This enabled the discrimination and mapping of CNVs, including single copy losses and gains, focal amplifications, and homozygous deletions within each cancer genome. Finally, we generated whole exome data for flow-sorted tumor populations from a subset of samples (*n* = 12) to increase the resolution for loci of interest and to incorporate somatic mutations and predicted neoepitopes into our genomic signatures. This combined approach provides high-resolution measures of TNBC genomes from ploidy, whole chromosome and chromosome arm level CNVs, focal amplicons, breakpoints, and homozygous deletions to the level of gene-specific insertion/deletions (indels) and mutations. These data provide a unique opportunity to assess the presence of individual and different classes of genomic lesions and to determine their association with the extent of PD-1 and PD-L1 expression in TNBC.

## Methods

### Clinical samples

TNBC samples were obtained under a Mayo Clinic protocol 2130–00 Cancer Tissue Study (principal investigator: B. Pockaj). This study was approved by Mayo Clinic institutional review board protocol 08–006579-08 Breast Cancer Clinical Genomics Project. The samples included 23 formalin-fixed paraffin-embedded (FFPE) and 32 fresh frozen tissues available for genomic analyses. Estrogen receptor (ER) and progesterone receptor (PR) were evaluated by standard American Society of Clinical Oncology/College of American Pathologists (ASCO/CAP) guidelines, and less than 1% of the cells stained for the receptors [12]. HER2-negative was defined by ASCO/CAP guidelines as staining by IHC of 0 or 1+ [13]. HER2 IHC of 2+ was further evaluated by fluorescence *in situ* hybridization (FISH) and deemed negative by standard ASCO/CAP guidelines. All biopsies in this study were from surgically resected tissue. These include the neoadjuvant-treated patients. All patients gave informed consent for collection and use of the samples. All tumor samples were histopathologically evaluated prior to genomic analysis. All research conformed to the Helsinki Declaration (https://www.wma.net/policies-post/wma-declaration-of-helsinki-ethical-principles-for-medical-research-involving-human-subjects/).

### Immunohistochemical staining

Whole tissue sections cut from FFPE tissue blocks were deparaffinized and rehydrated with serial passage through changes of xylene and graded ethanols. All slides were subjected to heat-induced epitope retrieval in Envision FLEX Target Retrieval Solution, High pH (Dako, Carpinteria, CA, USA). Endogenous peroxidase in tissues was blocked by incubation of slides in 3% hydrogen peroxide solution prior to incubation with primary antibody (anti-PD-L1, clone 22C3, Merck Research Laboratories, Palo Alto, CA, USA or anti-PD-1 clone NAT105, Cell Marque, Rocklin, CA, USA) for 60 min. Antigen-antibody binding was visualized via application of the FLEX+ polymer system (Dako) and application of 3, 3′ diaminobenzidine (DAB) chromogen (Dako). Stained slides were counterstained with hematoxylin and cover-slipped for review. For the scoring criteria, we used an established scoring system to report the PD-1 and PD-L1 expression levels in each sample [11]. Scoring of PD-1 and PD-L1 was conducted by a pathologist blinded to patient characteristics and clinical outcomes. A semi-quantitative 0–5 scoring system was applied: negative: 0; rare: 1 = individuated positive cells or only very small focus within or directly adjacent to tumor tissue; low: 2 = infrequent small clusters of positive cells within or directly adjacent to tumor tissue; moderate: 3 = single large cluster, multiple smaller clusters, or moderately dense diffuse infiltration within or directly adjacent to tumor tissue; high: 4 = single very large dense cluster, multiple large clusters, or dense diffuse infiltration; and very high: 5 = coalescing clusters, dense infiltration throughout the tumor tissue. Evaluations were relativized to the size of the tumor sample.

### Statistical analysis

Overall survival (OS) and disease-free survival (DFS) were estimated by using the Kaplan-Meier method, and differences were compared by using the log-rank test. Patients who were alive at the time of last follow-up were considered censored for OS, and patients without disease recurrence or death were considered censored for DFS. *P* values of less than 0.05 were considered statistically significant. Quantification of variance (Wilcoxon test) was performed for ploidy levels and CNV burden loads on tumors with high PD-1/PD-L1 expression versus tumors with low PD-1/PD-L1 expression. SAS version 9.4 (SAS Institute Inc., Cary, NC, USA) was used for analysis.

### Flow cytometry

Excess paraffin was removed from each FFPE sample with a scalpel from either side of 40- to 60-μm scrolls and processed in accordance with our published methods [14, 15]. We used a single 50-μm scroll from each FFPE tissue block to obtain sufficient numbers of intact nuclei for subsequent sorting and molecular assays. Frozen tissue biopsies were minced in the presence of NST buffer and 4′,6-diamidino-2-phenylindole (DAPI) in accordance with published protocols [14, 16, 17]. Nuclei from each sample were disaggregated and filtered through a 40-μm mesh prior to flow sorting with an Influx cytometer (Becton Dickinson, San Jose, CA, USA) with ultraviolet excitation and DAPI emission collected at more than 450 nm. DNA content and cell cycle were analyzed by using the MultiCycle software program (Phoenix Flow Systems, San Diego, CA, USA).

### Copy number analysis

DNAs from frozen tissue were treated with DNAse 1 prior to Klenow-based labeling. High-molecular-weight templates were digested for 30 min, whereas the smaller fragmented FFPE-derived DNA samples were digested for only 1 min. In each case, 1 μL of 10× DNase 1 reaction buffer and 2 μL of DNase 1 dilution buffer were added to 7 μL of DNA sample and incubated at room temperature and transferred to 70 °C for 30 min to deactivate DNase 1. Sample and reference templates were labeled with Cy-5 dUTP and Cy-3 dUTP, respectively, using a BioPrime labeling kit (Invitrogen, Carlsbad, CA, USA) in accordance with our published protocols [18]. All labeling reactions were assessed by using a Nanodrop assay (Nanodrop, Wilmington, DE, USA) prior to mixing and hybridization to Comparative Genomic Hybridization (CGH) arrays (Agilent Technologies, Santa Clara, CA, USA) for 40 h in a rotating 65 °C oven. All microarray slides were scanned by using an Agilent 2565C DNA scanner, and the images were analyzed with Agilent Feature Extraction version 11.0 using default settings. The array-based CGH (aCGH) data were assessed with a series of QC metrics and analyzed by using an aberration detection algorithm (ADM2) [19]. The latter identifies all aberrant intervals in a given sample with consistently high or low log ratios based on the statistical score derived from the average normalized log ratios of all probes in the genomic interval multiplied by the square root of the number of these probes. This score represents the deviation of the average of the normalized log ratios from its expected value of zero and is proportional to the height h (absolute average log ratio) of the genomic interval and to the square root of the number of probes in the interval. All aCGH data discussed in this publication have been deposited in the National Center for Biotechnology Information (NCBI) Gene Expression Omnibus (GEO) [20] and are accessible through GEO Series accession number GSE107764 (https://www.ncbi.nlm.nih.gov/geo/query/acc.cgi?acc=GSE107764).

### Fluorescent *in situ* hybridization

Home-brew *JAK2* DNA (clones RP11-980 L14, RP11-927H16, and CTD-2506A8) labeled with SpectrumOrange dUTP (Abbott Molecular, Abbott Park, IL, USA/Vysis Products) and commercially available chromosome 9 centromere (SpectrumGreen) provided by Abbott Molecular were combined as one probe set. The enumeration probe set was applied to individual slides, hybridized, and washed in accordance with published protocols [21].

### Whole exome sequencing

DNAs from each sorted tumor population and a patient-matched control sample were sequenced within the Mayo Clinic Medical Genome Facility (MGF) by using established protocols for whole exome analysis. Briefly, whole exon capture was carried out with Agilent’s SureSelect Human All Exon 71 MB version 6 kit; 500 ng of the prepped library is incubated with whole exon biotinylated RNA capture baits supplied in the kit for 24 h at 65 °C. The captured DNA:RNA hybrids are recovered by using Dynabeads MyOne Streptavidin T1 (Thermo Fisher Scientific, Waltham, MA, USA). The DNA was eluted from the beads and desalted by using purified Ampure XP beads (Beckman Coulter Life Sciences, Indianapolis, IN, USA). The purified capture products were amplified by using the SureSelect Post-Capture Indexing forward and Index polymerase chain reaction (PCR) reverse primers (Agilent Technologies) for 12 cycles. Libraries were loaded onto paired-end flow cells at concentrations of 4–5 pM to generate cluster densities of 600,000–800,000/mm^2^ by using the Illumina cBot and HiSeq Paired-end cluster kit version 3 (Illumina, San Diego, CA, USA). The flow cells are sequenced as 101×2 paired-end reads on an Illumina HiSeq 2500 or 4000 by using TruSeq SBS sequencing kit version 3 and HiSeq data collection version 1.4.8 software. Base-calling was performed by using Illumina’s RTA version 1.12.4.2.

### Variant calling and annotation

We started with aligned tumor and germline data (in bam format) for each patient. We used VarScan2 (version 2.3.9) [22] available on a high-performance cluster computing environment to call tumor-specific variants. We applied a minimum coverage of 10 reads in normal and tumor to call somatic variants, a minimum variant frequency of 0.08 to call a heterozygote, and a somatic *P* value of 0.05 as a threshold to call a somatic site. We further filtered the SNP calls to remove those near indel positions and also removed likely false positives associated with common sequencing- and alignment-related artifacts [23]. We used the variant effect predictor tool [24] with ensemble transcript versions for the hg19 reference genome to generate fasta sequences for a range of flanking amino acids (7, 8, 9, and 10 bp) on each side of the mutated amino acid to generate 15, 17, 19, and 21 amino acid sequences, respectively, to be used in the inference of neoepitopes (see below). We also annotated the variants functionally by using Annovar [25] with hg19 reference genome.

### HLA typing

We used the POLYSOLVER (POLYmorphic loci reSOLVER) algorithm [26] to infer the HLA types present in each patient by using the germline (normal) whole exome sequencing data. The method employs a Bayesian classifier and selects and aligns putative HLA reads to an imputed library of full-length genomic library of HLA alleles. We included three major histocompatibility complex (MHC) class I (HLA-A, -B, and -C) genes for HLA typing.

### Neoepitope generation and filtering

We generated all possible 8mers, 9mers, 10mers, and 11mers (neoepitopes), including the mutant amino acid, using a sliding window with the mutant amino acid at each possible position. To infer the binding of each potential neoepitope to the patient-specific HLA alleles, we used the Immune Epitope Database (IEDB) prediction method from the IEDB [27] for all possible combinations of HLAs and neoepitopes. Our final set included only epitopes with a binding affinity (ann_ic50) of less than 500 nM for the patient-specific HLA alleles.

## Results

In total, 55 TNBC cases were screened for PD-1 and PD-L1 expression by IHC (Additional file 1: Figure S1). One of these failed because of low tumor content in the tissue sample. Of the remaining 54 cases with IHC data, 39 were treatment-naïve at the time of resection. Biopsies from 48 of the 55 TNBCs were available for flow sorting. These included 32 of the 39 treatment-naïve cases. However, the biopsy for one case had only a single diploid population that was copy number–neutral. In addition, we sorted and obtained genomic data for the surgical samples of the 16 available cases that received neoadjuvant therapy. Fifteen of these 16 had corresponding IHC data. Thus, our final results include combined IHC tissue analyses and genomic data of flow-sorted tumor populations for 31 treatment-naïve and 15 treatment-positive TNBCs. In addition, we sequenced the exomes of flow-sorted tumor populations from six treatment-naïve and six treated cases and the transcriptomes of whole biopsies from three treatment-naïve and six treated cases with IHC and CNV profiles of interest.

### PD-1 and PD-L1 expression patterns

**Fig. 1:**
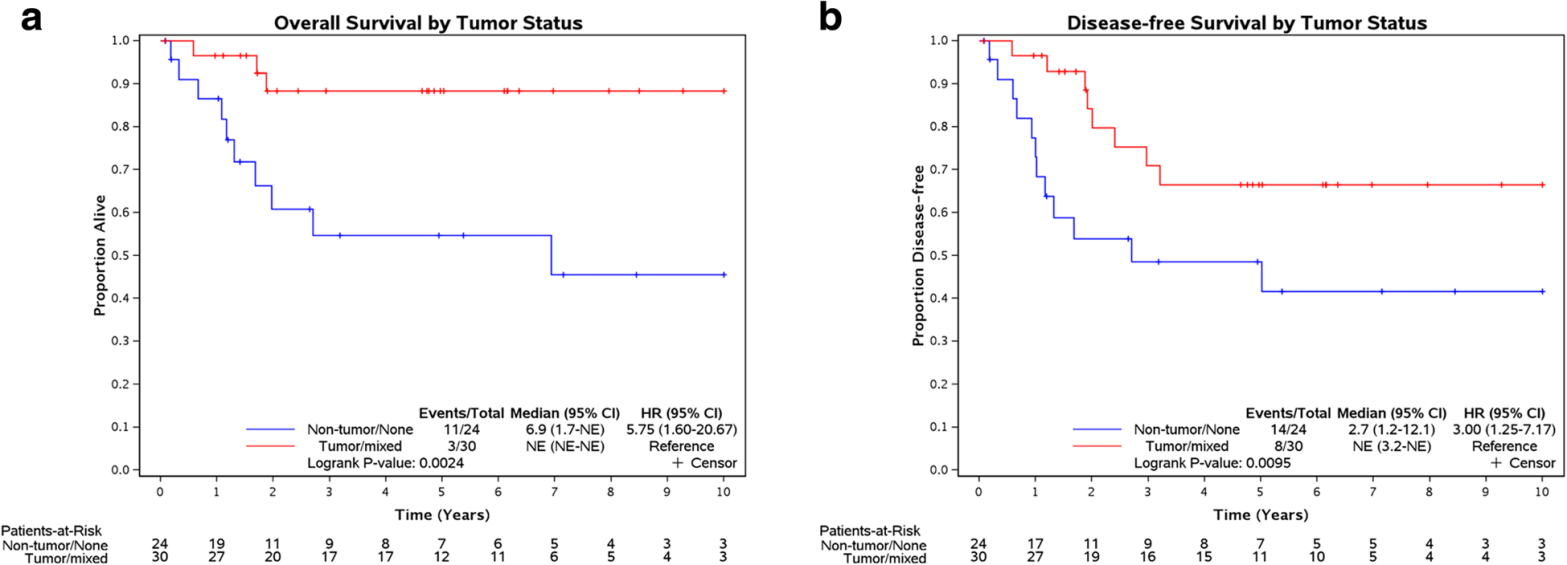
Overall survival, disease-free survival, and expression of PD-L1. **a** Overall survival and (**b**) disease-free survival were estimated by using the Kaplan-Meier method and differences were compared using the log-rank test. Patients who were alive at the time of last follow-up were considered censored for overall survival. *P* values of less than 0.05 were considered statistically significant. SAS version 9.4 (SAS Institute Inc.) was used for analysis. Abbreviations: *CI* confidence interval, *HR* hazard ratio, *NE* not estimated

There was a broad range of expression for both proteins in the 54 evaluable cases (Table 1). Eleven of 54 (20.0%) and 20 out of 54 (37%) of TNBCs had high (IHC score of 4) or very high (IHC score of 5) staining for PD-1 and PD-L1, respectively. The 11 cases with elevated expression of PD-1 had matching increases of PD-L1. Strikingly, PD-L1 expression in the 15 out of 20 (75%) cases with IHC scores of at least 4 was almost exclusively on the surfaces of tumor cells. In contrast, nine out of 54 (17%) and seven out of 54 (13%) TNBCs had negative (IHC score of 0) or rare (IHC score of 1) staining for PD-1 and PD-L1, respectively. The PD-L1 staining had a broader range compared with PD-1 with three negative cases in addition to the 20 cases with an IHC score of at least 4. Notably, 15 of these PD-L1 elevated expression cases, including the eight with a maximum IHC score of 5, were treatment-naïve. Despite the range of PD-L1 expression, there were no significant correlations with the level of expression on tumor or non-tumor cells and OS or PFS in our cohort. In contrast, the presence of any PD-L1 expression (IHC score of 1–5) on tumor cells was a significant correlate of OS (log-rank *P* value: 0.0024) and of DFS (log-rank *P* value: 0.0095) (Fig. 1).

**Table 1:** PD-1 and PD-L1 expression in triple-negative breast cancer

IHC score	PD-1	PD-L1
Negative 0	0	3 (2)
Rare 1	9 (5)^a^	4 (2)
Low 2	17 (13)	15 (11)
Moderate 3	17 (12)	12 (9)
High 4	10 (8)	12 (7)
Very high 5	1 (1)	8 (8)

^a^Treatment-naïve cases

Abbreviation: *IHC* immunohistochemistry

### Genomic lesions in resected TNBCs

Aneuploid peaks were detected and then sorted from 39 out of 48 (81%) available biopsies, providing pure tumor populations for genomic analyses (Additional file 2: Figure S2 and Additional file 3: Figure S3). In eight out of nine biopsies without an aneuploid peak, we sorted and subsequently confirmed tumor content in the 4N(G_2_/M) fraction. The remaining sample was diploid only by flow cytometry and copy number–neutral by CNV analysis. Although the tumor ploidies varied from diploid to hypertetraploid, there was no association with high (*n* = 17) or low (*n* = 6) expression levels of PD-L1 (Wilcoxon rank-sum test, *P* = 0.31).

The DNAs from each sorted population of interest were interrogated with CGH arrays to confirm the tumor content and to provide a CNV profile of each tumor genome. We used the ADM2 step gram algorithm to distinguish aberrant copy number intervals and map their boundaries in each flow-sorted tumor population. There was extensive heterogeneity in the CNV profiles of the TNBC cases. The number of aberrant intervals varied from less than 10 to more than 80 in each TNBC genome. However, there was no association with CNV burden and PD-L1 expression (Wilcoxon rank-sum test, *P* = 0.92). The intervals included whole chromosomes, chromosome arms, and interstitial aberrations in the TNBC genomes. Of significant interest were focal CNVs, including high-level amplicons and homozygous deletions that recurrently targeted oncogenic pathways associated with TNBCs. At least one focal amplicon defined by log_2_ ratios of more than 1 and genomic boundaries of less than 10 Mb was identified in 43 out of 47 (91%) of the TNBC genomes. These included recurring focal amplicons targeting oncogenic drivers *EGFR* (5/47), *JAK2* (9/47), *AKT2* (3/47), *FGFR2* (3/47), and *MYC* (9/47) (Table 2). Amplified copies of *MYC* were present in 10 additional cases where the amplicon extended beyond 10 Mb, including six cases with whole 8q gains. The *JAK2* copy number status of our TNBC cohort, including both gains and losses of 9p24.1, was validated with a FISH assay (Additional file 4: Figure S4, Additional file 5: Figure S5, and Additional file 6: Figure S6) [28]. In addition, our use of pure flow-sorted samples revealed multiple homozygous deletions, ADM2-defined intervals with log_2_ ratios of not more than −3.0, in these samples. These include deletions targeting known tumor suppressor genes (*CDKN2A*, *RB1*, *PTEN*, *ARID1B*, *JAK1*, and *BRIP1*) as well as unique targets (*EPS8*, *GRB10*, *EIF4G3*, *STK4*, and *RBM9*) in TNBC.

**Table 2:** Driver amplicons

Sample	MYC	EGFR	AKT2	FGFR2	JAK2
TNBC-2	+				
TNBC-4	+++++				++
TNBC-7	++			+++++	
TNBC-8	++				
TNBC-11	++				++++
TNBC-13			+++	++	
TNBC-16	++				
TNBC-17	+				
TNBC-18	++	++			+++
TNBC-19	++				
TNBC-20	++				
TNBC-23	++		++		
TNBC-27	++				
TNBC-29					+++
TNBC-30	++				
TNBC-36	++			++	
TNBC-39	+	++			
TNBC-43	++				++
TNBC-44		+++++			++
TNBC-47	+++				
TNBC-49			+++		++
TNBC-50		+++++			
TNBC-51	++				+
TNBC-53	++	++			++

+++++: log_2_ ratio > 4

++++: log_2_ ratio > 3

+++: log_2_ ratio > 2

++: log_2_ ratio > 1

+: log_2_ ratio ≥ 1

The combined IHC and genomic data were used to investigate associations between PD-1 and PD-L1 staining patterns and genomic aberrations of interest. These include gene and signaling pathway-specific lesions and measures of genomic instability across the genome. Of significant interest was the identification of recurring genomic lesions and profiles in those TNBCs with high levels of PD-L1 on the surface of tumor cells.

### CNVs and treatment-naïve TNBCs with increased PD-L1 expression

**Fig. 2:**
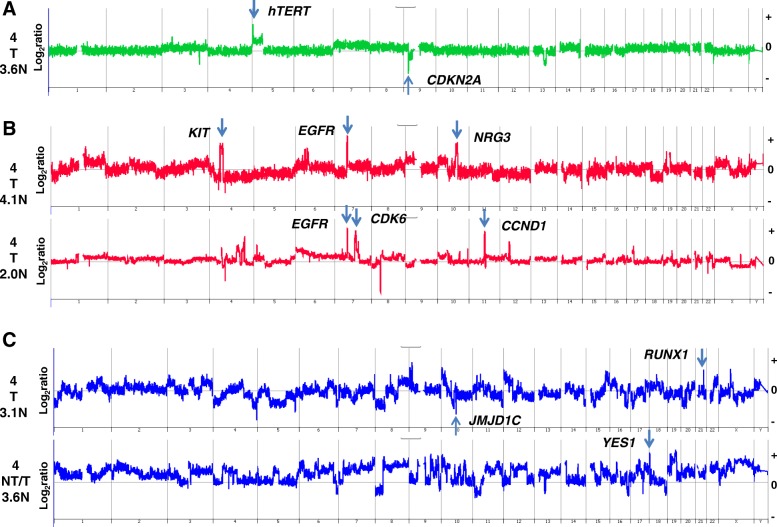
Whole genome CNV profiles of chemoradiation-naïve TNBCs with high levels of PD-L1 expression on tumor cell surfaces. TNBCs with high levels (IHC score ≥4) of PD-L1 included cases with (**a**) low number of CNVs (TNBC-33), **b** multiple focal high-level amplicons targeting known driver genes (TNBC 44 and TNBC-50), and (**c**) genomes with high CNV burdens (TNBC-14 and TNBC-23). PD-L1 IHC scores and location (*T* tumor cells, *T/NT* tumor plus non-tumor cells) as well as the DNA ploidy (N) of each TNBC are presented. The X and Y axes in the Comparative Genomic Hybridization plots represent chromosome and log_2_ ratios for each TNBC. Abbreviations: *CNV* copy number variant, *IHC* immunohistochemistry, *TNBC* triple-negative breast cancer

Whole genome CNV data were derived for 12 out of 15 treatment-naïve TNBCs with high (IHC score of 4) or very high (IHC score of 5) PD-L1 expression. The genomes of these TNBCs had a broad range of total number of CNVs, including focal amplicons targeting oncogenic drivers (Table 3). We focused on five of these treatment-naïve cases with combined IHC and CNV data to initially investigate the association of CNV burden and candidate driver amplicons with PD-L1 expression patterns (Fig. 2). One of the five cases had an aneuploid genome with a relatively simple CNV profile consisting of gain of chromosome 7, gain of chromosome 5p with an additional interstitial gain of p15.33-p15.32, a loss spanning 13q21.31-q22.2, and a homozygous deletion targeting *CDKN2A* (Fig. 2a). In contrast, two cases—one diploid and the other hypertetraploid by flow cytometry—had high-level (log_2_ ratio >4.0) focal amplification of *EGFR* with additional unique high-level focal amplicons targeting *CDK6* and *CCND1*, and *KIT* and *CCNE1*, in each of the tumors (Fig. 2b). RNA-seq analysis of the latter case confirmed the high expression of *EGFR*, *KIT*, and *CCNE1* with the presence of the corresponding high-level focal amplicons. The two additional treatment-naïve cases were aneuploid by flow cytometry and had extensive numbers of CNVs throughout their genomes (Fig. 2c). These included high-level amplicons targeting *RUNX1* and *YES1* oncogenes and a homozygous deletion of *JMJD1C*, a demethylase that regulates the BRCA1-mediated DNA damage response pathway [29].

**Table 3:** Focal amplicons and PD-L1 expression

Sample	Ploidy	EGFR	JAK2	AKT2	MYC	FGFR2	PD-L1 (IHC)	Neoadjuvant
TNBC-6	4.0						5 T	–
TNBC-9	4.0						5 T	–
TNBC-14	3.1		+++		++		5 T	–
TNBC-21	2.8						5 T	–
TNBC-24	2.5						5 T	–
TNBC-33	3.6						5 T	–
TNBC-37	NB						5 T	–
TNBC-40	3.4						4 NT	–
TNBC-16	3.2				++		4 T	–
TNBC-23	3.6			++	++		4 T/NT	–
TNBC-44	4.1	+++++	++				4 T	–
TNBC-46	ND						4 T/NT	–
TNBC-50	2.0	+++++					4 T/NT	–
TNBC-55	3.0		++		++		4 NT	–
TNBC-5	3.0				++		4 T	+
TNBC-8	3.3				++		4 NT	+
TNBC-29	4.0		++				4 T	+
TNBC-36	2.0				++	++	4 T/NT	+
TNBC-43	3.2				+++		4 T/NT	+
TNBC-18	4.2	++	+++		++		1 NT	–
TNBC-30	3.1				++		1 NT	–
TNBC-4	3.2		++		+++++		1 NT	+
TNBC-7	3.8				++	+++++	1 NT	+
TNBC-51	3.5		++		++		0	+
TNBC-20	3.4				++		0	–
TNBC-56	ND						0	–

+++++: log_2_ ratio > 4

+++: log_2_ ratio > 2

++: log_2_ ratio > 1

Abbreviations: *IHC* immunohistochemistry, *NB* no tumor in biopsy, *ND* not done, *NT* non-tumor cells, *T* tumor cells

### CNVs and treatment-naïve TNBCs with reduced PD-L1 expression

**Fig. 3:**
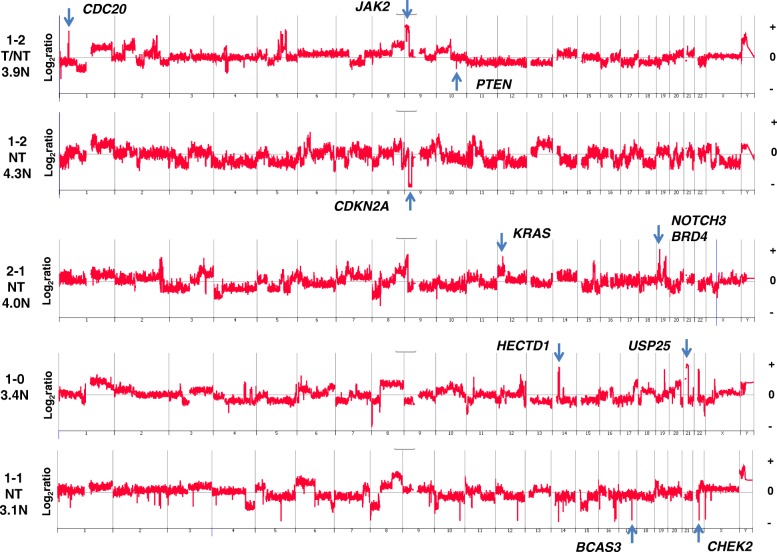
Whole genome CNV profiles of chemoradiation-naïve TNBCs with low levels of PD-L1 and PD-1 expression. TNBCs with low levels (IHC score ≤2) of PD-1 and PD-L1 and high levels of CNVs throughout their genomes (top to bottom: TNBCs, 11, 17, 18, 20, and 30). PD-L1 staining location (*NT* non-tumor cells, *T/NT* tumor plus non-tumor cells) as well as the DNA ploidy (N) of each TNBC are presented. The X and Y axes in the Comparative Genomic Hybridization plots represent chromosome and log_2_ ratios for each TNBC. Abbreviations: *CNV* copy number variant, *IHC* immunohistochemistry, *TNBC* triple-negative breast cancer

Four of the seven TNBCs with negative (IHC score of 0) or rare (IHC score of 1) PD-L1 staining were treatment-naïve (Table 1). Two additional treatment-naïve cases with low (IHC score of 2) PD-L1 expression had a combined IHC score of only 3, suggesting low activity of the PD-1–mediated checkpoint. Genomic analysis of five of these six low-activity cases identified distinct CNVs, including focal amplicons targeting known oncogenes *KRAS* and *JAK2* as well as homozygous deletions of variable sizes targeting tumor suppressor genes, including *CDKN2A* and *PTEN* (Fig. 3). However, there were no significant differences in the prevalence of these CNVs between TNBCs with low or high PD-L1 expression (Table 3).

### DNA repair pathway lesions

There were 13 TNBCs with elevated numbers (>50) of intrachromosomal CNV aberrations often seen in *BRCA* mutant tumors. IHC data were obtained for 12 out of 13 of these cases. We identified DNA mutations or homozygous deletions in DNA repair pathway genes in nine out of 12 with IHC results (Table 4). Strikingly, one case had a homozygous deletion in *MLH3* and another had a somatic *MSH2*^F289C^ mutation. Notably, the whole exome data of the *MSH2* mutant case confirmed the MSI status of the tumor cells. However, both neoadjuvant-treated cases had low or moderate expression of PD-1 and PD-L1, the latter exclusive to the non-tumor cells. In seven additional high CNV burden cases with IHC data, we identified homozygous deletions of *CHEK2*, *BRIP1*, and *DCLRE1C* and mutations in *BRCA1*, *FBXW7*, *PRKDC*, and *ALKBH5*. Two of these—*BRCA1*^mut^ and *PRKDC*^Q75R^—had high PD-L1 expression on the surface of tumor cells whereas the other three had rare or low expression on non-tumor cells. The genetic basis for elevated numbers of CNVs was not determined in three cases profiled by CGH only.

**Table 4:** Triple-negative breast cancers with high copy number variant burden^1^

Sample	Gene lesion	PD-1	PD-L1	Neoadjuvant
TNBC-8	MSH2^F^^289C^	2	4 NT	+
TNBC-53	MLH3^−/−^	3	3 NT	+
TNBC-30	CHEK2^−/−^	1	1 NT	–
TNBC-31	BRIP1^−/−^	3	2 NT	–
TNBC-24	BRCA^mut^	4	5 T	–
TNBC-27	FBXW7^S398F^	2	3 NT	+
TNBC-29	PRKDC^Q75R^	2	4 T	+
TNBC-17	ALKBH5^P303Q^	1	2 NT	–
TNBC-36	DCLRE1C^656_671del16^	3	4 T/NT	+
TNBC-54	TBD	2	2 T/NT	+
TNBC-51	TBD	1	0	+
TNBC-23	TBD	3	4 T/NT	–

^1^>50 intrachromosomal copy number variants

Abbreviations: *NT* non-tumor cells, *T* tumor cells, *TBD* to be determined, *TNBC* triple-negative breast cancer

### PTEN/PIK3CA

**Fig. 4:**
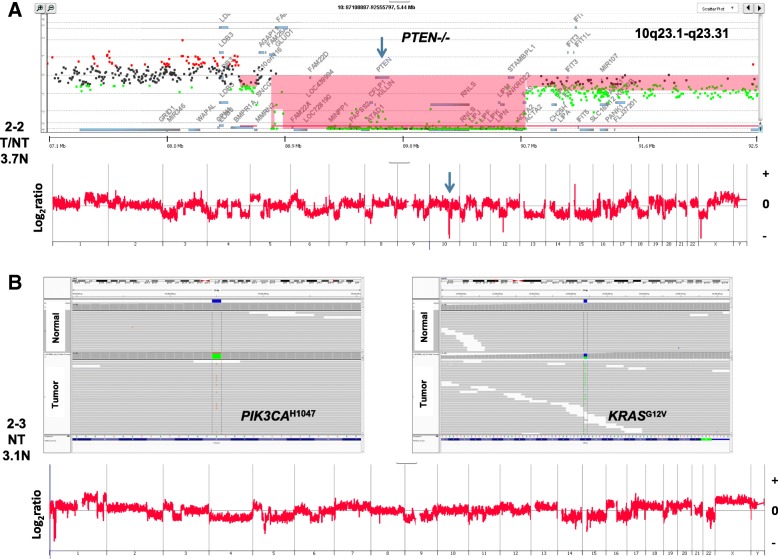
AKT pathway-specific lesions in TNBC genomes. **a** Whole genome (bottom panel) and locus-specific (top panel) mapping of a PTEN homozygous deletion in TNBC-1. Red shaded area denotes ADM2-defined homozygous deletion. **b** IGV views (top panels) of activating *PIK3CA*^H1087R^ and *KRAS*^G12V^ mutations in genome (bottom panel) of TNBC-34. Abbreviations: *NT* non-tumor cells, *T/NT* tumor plus non-tumor cells, *TNBC* triple-negative breast cancer

Homozygous deletions targeting *PTEN* were detected in six of the 46 (13%) cases profiled by CGH and IHC (Table 5, Fig. 4a). Three of these six were treatment-naïve TNBCs. We sequenced the exomes of 12 of the TNBCs, including 11 with intact *PTEN*, and detected an activating *PIK3CA*^H1047R^ mutation in the aneuploid genome of another treatment-naïve tumor (Fig. 4b). Thus, in seven cases, the genomic results support an active AKT signaling context. The expression of PD-1 was rare or low in all seven cases while PD-L1 was low or moderate in six out of seven and high in one case. However, in all cases, the expression of PD-L1 was noted almost exclusively on non-tumor cells. This is in contrast to reports that loss of *PTEN* and activated AKT signaling upregulates PD-L1 and leads to its increased tumor cell surface expression in TNBC and other solid tissue tumors [30–32].

**Table 5:** PD-1 and PD-L1 expression

TNBC	PD-1	PD-L1	Tumor/Non-tumor	PTEN/PIK3CA	Neoadjuvant
TNBC-1	2	2	T/NT	PTEN^−/−1^	–
TNBC-8	2	3	NT	PTEN^−/−^	+
TNBC-10	2	3	T/NT	PTEN^−/−^	+
TNBC-11	1	2	T/NT	PTEN^−/−^	–
TNBC-34	2	3	NT	PIK3CA^H1047R^	–
TNBC-35	1	2	NT	PTEN^−/−^	+
TNBC-55	2	4	NT	PTEN^−/−^	–

^1−/−^: Homozygous deletion

Abbreviations: *NT* non-tumor cells, *T* tumor cells, *TNBC* triple-negative breast cancer

### Mutation load and predicted neoepitopes

The number of non-conserved somatic mutations detected in the exomes of the 12 flow-sorted TNBCs ranged from 16 to 146 (Table 6). The number of predicted neoepitopes varied from 69 to 1368. Notably, MSI^+^ TNBC-8 had an elevated number of non-conserved mutations and of predicted neoepitopes. Strikingly, TNBC-36 and TNBC-17, both microsatellite-stable (MSS) and *BRCA*^wt^, had the highest mutation loads and numbers of predicted neoepitopes. We detected a 16-bp indel in DNA Cross Link Repair 1C (*DCLRE1C*) and a non-conserved *ALKBH5* mutation in these two cases. The former, also known as Artemis, plays an essential role in VDJ recombination and may mediate double-strand DNA repair, whereas *ALKBH5* is an RNA demethylase that has been implicated in direct DNA repair [33, 34]. However, PD-L1 staining was regionally high in TNBC-36 and low in treatment-naïve TNBC-17.

**Table 6:** Mutation load and neoepitopes

TNBC case	Number of neoepitopes (ann < 500)	Non-synonymous mutations	Synonymous mutations	Stop gain	Stop loss
TNBC-27	129	23	19	1	0
TNBC-29	413	61	30	3	0
TNBC-44	388	50	45	5	0
TNBC-2	70	21	5	1	0
TNBC-49	69	54	54	0	0
TNBC-8	557	64	29	4	0
TNBC-25	148	16	9	0	0
TNBC-34	243	33	20	2	0
TNBC-38	187	26	25	4	0
TNBC-47	88	41	13	1	0
TNBC-36	1368	137	55	9	0
TNBC-17	1116	137	65	8	1

Abbreviation: *TNBC* triple-negative breast cancer

## Discussion

The expression of PD-L1 on the surfaces of tumor cells has been used in clinical trials to identify and enrich for patients who will benefit from immunotherapy [35–37]. However, clinical benefit has also been seen in subsets of patients with low tumor cell PD-L1 expression [38]. The expression of PD-1 and PD-L1 can vary over time and within regions of tumors of interest. Thus, the timing of a biopsy relative to treatment and the extent of tissue and genomic heterogeneity within tumors may affect the sensitivity and specificity of IHC-based biomarkers. Furthermore, the multiple PD-1 and PD-L1 antibodies available for clinical studies and the variable scoring thresholds applied have limited the development of IHC-based prognostic assays.

Genomic-based biomarkers would provide an alternative or complementary approach to identify those patients who may benefit from or be refractory to emerging immunotherapies. Here, we used flow-sorted tumor samples from well-annotated surgically resected TNBCs for genomic analyses. We applied validated PD-1 and PD-L1 antibodies and a standardized IHC scoring system to characterize the expression patterns in these primary TNBCs, including 39 neoadjuvant treatment-naïve cases. There was a broad range of PD-1 and PD-L1 expression in our cohort with expression of PD-L1 noted exclusively on either tumor or non-tumor cells or on both within the tissue. However, our combination of IHC staining with genomic profiles of flow-sorted tumor populations in our cohort of surgically resected TNBCs represents a unique data set to test current hypotheses related to genomic lesions and signatures associated with expression of PD-1 and PD-L1.

### Aneuploidy

Aneuploidy can be defined by a number of measures. DNA content flow cytometry discriminates differences in total DNA between tumor and coexisting non-tumor cells in samples of interest. Our DAPI-based flow cytometry assays have coefficients of variation (CVs) of 5–10%, allowing discrimination of nuclei with at least 2.2 N DNA content from diploid in solid tumor biopsies. The widths of the CVs for DNA content histograms can vary with the quality of biopsies notably with archived FFPE samples. This can affect the purity and yield of sorted tumor populations. However, careful placing of sorting gates can separate pure tumor and non-tumor populations even from suboptimal samples (Additional file 2: Figure S2 and Additional file 3: Figure S3). In contrast, cytogenetics assesses ploidy by the presence or absence of chromosomes with the resolution of a single chromosome. Thus, cells with only an extra copy of a smaller chromosome (e.g., chromosome 21), which may not be detected as a difference in total DNA in our flow cytometry assay, are classified as aneuploid by karyotype-based methods. Alternatively, tumors may contain multiple CNV regions and chromosome imbalances of gains and losses that result in an average “diploid by flow” DNA content. An additional method is to estimate DNA content from genomic data of bulk tumors [39]. Notably, recent reports of PD-1/PD-L1 checkpoint activation estimated tumor aneuploidy as the burden of whole chromosome and chromosome arm aberrations from whole exome sequencing data [6]. In total, 39 out of 48 (81%) evaluable TNBCs in our study were aneuploid by flow cytometry. The ploidies of these cases ranged from 2.3 N to 5.1 N. Eight of the remaining nine cases were sorted as diploid/tetraploid fractions and then confirmed to be aneuploid at the genome and chromosome level by CNV analysis. However, despite the range of ploidies and the variable numbers of chromosomal aberrations, we did not observe any correlation of tumor DNA content with IHC staining for either PD-1 or PD-L1.

### CNVs

The use of flow-sorted tumor populations for CNV analysis enabled the identification of known driver lesions, including high-level focal amplicons targeting *EGFR*, *JAK2*, *AKT2*, *MYC*, and *FGFR2*, as well as homozygous deletions of both well-established *PTEN*, *CDKN2A*, *ARID1B*, *GRB10*, *BRIP1*, *JAK1*, and *RB1*, and unique *RBM9*, *CEBPG*, and *EIF4G3* TNBC tumor suppressor genes. The two treatment-naïve cases with the highest level (log_2_ ratio >4.0) focal EGFR amplicons had uniform high staining of PD-L1 on the tumor cell surfaces (Fig. 2). However, this pattern was not observed on three additional cases with moderate-level (1.0 < log_2_ ratio < 4.0) EGFR amplicons. Thus, the level of EGFR amplification and expression may need to exceed a threshold to elicit elevated PD-L1 levels. The two highly EGFR-amplified cases also contained high-level focal amplicons targeting other well-known oncogenic pathways (Fig. 2), suggesting that additional co-occurring genomic lesions may contribute to PD-L1 overexpression in TNBC tumor cells.

Multiple studies have interrogated clinical biopsies obtained before and after immunotherapy with the aim of identifying recurring genomic aberrations that correlate with response. Notably, loss of heterozygosity (LOH) of immune-responsive alleles has been reported to be associated with loss of clonal T cells in patients with non-small cell lung cancer who relapsed [40]. In addition, disruption of HLA alleles has been linked to loss of immunogenicity and poor outcomes [26, 41, 42]. The highly aneuploid nature of TNBCs at both the ploidy and chromosome level disrupts the ratio of alleles throughout the genome. Thus, LOH, which can be driven by ongoing genomic instability in aneuploid genomes, may have significant impact on immune signatures of TNBC. Additionally, a CRISPR screen identified a series of genes that are essential for effector function of CD8^+^ T cells targeting melanoma cells [43]. We noted mutations and CNVs in multiple “hits” from this screen, including *MYO1B*, *VHL*, and *ARID2*, in our cohort. Of significant interest will be to apply our flow sorting–based genomic analyses to biopsies of relapsed TNBCs from immunotherapy trials.

### PDJ amplicon

**Fig. 5:**
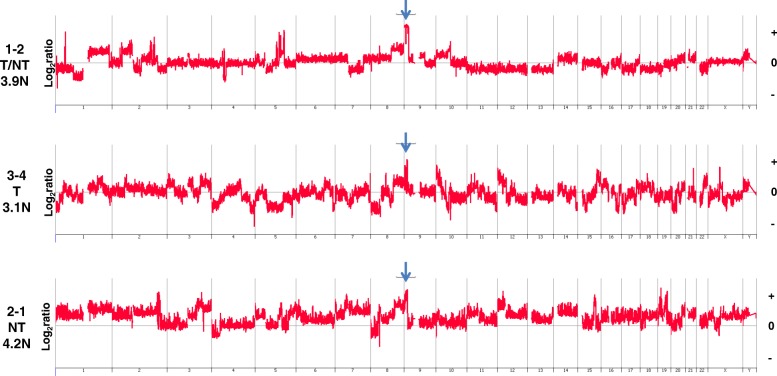
PDJ^+^ TNBC genomes. Treatment-naïve TNBCs (TNBC-11, TNBC-14, and TNBC-18) with high-level PDJ amplicon (blue arrows) (log_2_ ratio >2). PD-1 and PD-L1 IHC scores and location (*NT* non-tumor cells, *T* tumor cells, *T/NT* tumor plus non-tumor cells) as well as the DNA ploidy (N) of each TNBC are presented. The X and Y axes in the Comparative Genomic Hybridization plots represent chromosome and log_2_ ratios for each TNBC. Abbreviations: *IHC* immunohistochemistry, *TNBC* triple-negative breast cancer

Copy number increases of the PD-L1 locus have been reported in a variety of tumors [44–47]. We and subsequently others have shown that a 9p24.1 amplicon targeting JAK2 and PD-L1 (PDJ amplicon) is enriched in TNBC [15, 48]. Notably, our study of flow-sorted tumor populations confirmed that this PDJ amplicon is present in chemoradiation-naïve resected cases and is associated with transcriptional upregulation of both genes [15]. However, the functional significance of PDJ amplification on immune regulation and response to checkpoint blockade is not known. There were three treatment-naïve TNBCs with a high-level (log_2_ ratio >2.0) 9p24.1 PDJ amplicon that included JAK2 and PD-L1 (Fig. 5) [15]. Only one of these three had a corresponding increase in PD-L1 on the tumor cell surface but with a striking difference in staining intensity between the undifferentiated regions of the tumor (IHC score of 5) and those that were differentiated (IHC score of 0). Expression of PD-L1 can be induced by interferon-gamma (IFN-γ) in multiple cell types, including TNBC [49, 50]. In our preliminary studies, we have observed that in TNBC cell lines with 9p24.1 copy number gain, PD-L1 expression was markedly and rapidly inducible by low-dose IFN-γ in a copy number–dependent manner, mimicking an *in situ* inflammatory response. Although RNA interference (RNAi)-mediated knockdown of JAK2 in TNBC cells did not affect constitutive PD-L1 expression, it did block IFN-γ–induced PD-L1 expression ([51] Chen et al., in press 2018). Notably, this was specific to cells with CNV gains of 9p24.1. Thus, the PDJ amplicon is associated with a dynamic IFN-inducible PD-L1 expression on tumor cells.

In contrast to JAK2 amplification, we also identified a 1.8-Mb homozygous deletion at 1p31.3 that included the *JAK1* locus in a post-neoadjuvant–treated case (Additional file 7: Figure S7). This TNBC had rare PD-1 expression in non-tumor cells and was negative for PD-L1 expression. Given the association of *JAK1* mutation and loss of the wild-type allele with an acquired resistance to PD-1 blockade in melanoma, this homozygous mutation may create the similar clinical context in TNBC [42, 52].

### PTEN/PIK3CA

In addition to targeted amplification, homozygous deletions and somatic mutations may activate oncogenic signaling pathways. Notably, there were six cases with homozygous deletions within *PTEN* and a seventh with a common activating mutation of *PIK3CA* [53, 54]. The latter also had a *KRAS*^G12V^ mutation (Fig. 4). Strikingly, all seven of these TNBCs lacked elevated expression of PD-L1 on tumor cell surfaces (Table 5). This is in contrast to studies of loss of *PTEN* and activation of PI3K-AKT signaling causing elevated expression of PD-L1 on the surface of most cancer cells within glioblastomas [31]. Furthermore, knockdown of *PTEN* in model systems has been reported to increase expression of PD-L1 and its appearance on the TNBC cell surface [30]. Thus, it has been hypothesized that targeting the PI3K signaling pathway in TNBC may provide additional benefit for patients treated with immunotherapy. However, our current data, which discriminate homozygous from partial *PTEN* copy number loss in flow-sorted tumors, suggest that further clinical studies applying precision genomics and well-annotated clinical samples are needed to define the role of PI3K-AKT signaling in the immune signatures and responses of TNBCs.

### DNA repair lesions

Twelve of thirteen TNBC genomes with elevated numbers of interstitial CNVs, a context associated with DNA repair deficiencies, had matching IHC data (Table 1). One case had a pathogenic *BRCA1* mutation that was detected prior to surgery in a clinical laboratory. Three additional cases had homozygous deletions in genes with known roles in DNA repair pathways. We sequenced the exomes of five of the remaining seven TNBCs with this DNA repair deficiency signature to identify additional mediators of this clinical phenotype. Combined homozygous deletions and mutations accounted for 10 out of 13 TNBCs with this CNV signature. Strikingly, two cases also had lesions in mismatch repair genes, *MLH3* and *MSH2*. In the latter case, the MSI^+^ status was confirmed by whole exome next-generation sequencing. The expression of PD-L1 was exclusive to the non-tumor cells in both of these cases. Given the reports of striking responses of MSI^+^ tumors to anti-immune checkpoint therapy, additional studies are needed to determine the association of MSI status with PD-1 and PD-L1 expression in these highly aberrant TNBCs.

The mutation load varied across the 12 TNBCs whose exomes were sequenced. Strikingly, TNBC-36 and TNBC-17 had over twice as many mutations and predicted neoepitopes, 1368 and 1116, respectively, as MSI^+^ TNBC-8 (Table 6). Both cases also had elevated CNV loads with mutations in *DCLRE1C* and *ALKBH5* (Additional file 8: Figure S8). Despite these shared genomic features, TNBC-36 had regionally high levels of PD-L1 expression on tumor cells while TNBC-17 had low PD-L1 expression on non-tumor cells.

## Conclusions

PD-L1 expression on tumor cell surfaces correlated with improved OS and DFS in resected TNBCs. However, PD-L1 expression was highly variable in TNBCs even with genomic contexts such as MSI^+^ and high CNV burden that are associated with clinical benefit from immune checkpoint inhibition. Therefore, given the complexity of TNBC genomes, simple correlations of genomic lesions with presence and levels of PD-1 and PD-L1 proteins may not provide robust predictive markers. For example, EGFR amplicons need to be well defined and placed in the context of other co-occurring aberrations. Thus, incomplete genomic and CNV profiles such as targeted panel sequences of bulk tumor samples may not provide the resolution needed to develop and validate solid tumor biomarkers for immunotherapies. Although larger studies are needed to fully develop our observations, there was a clear lack of association between pathogenic lesions targeting the PI3K-AKT pathway and increased expression of PD-L1 on tumor cell surfaces. Future studies will incorporate the location and the level of activity of tumor-infiltrating lymphocytes within TNBC tissues. In addition, T-cell receptor sequencing will prioritize tumor-specific neoepitopes identified in samples of interest. Our use of flow-sorted clinical samples will provide the resolution needed to resolve the association of genomic lesions with immune signatures and clinical responses for patients with TNBC.

## Additional files

Additional file 1: **Figure S1.** Workflow and analyses of TNBC cohort. Fifty-five resections were screened for PD-1 and PD-L1 expression with IHC. Biopsies from 48 resections were flow-sorted and profiled for CNVs. Combined IHC and CNV data were obtained from 46 cases in this study. Abbreviations: *CNV* copy number variant, *IHC* immunohistochemistry, *TNBC* triple-negative breast cancer. (PPTX 74 kb)

Additional file 2: **Figure S2.** Flow-sorting formalin-fixed paraffin-embedded (FFPE) TNBC tissue samples. DNA content analysis of diploid and aneuploid populations flow-sorted from FFPE TNBC tissues. DNA content and cell cycle were analyzed by using the MultiCycle software program (Phoenix Flow Systems, San Diego, CA, USA). Abbreviation: *TNBC* triple-negative breast cancer. (PPTX 603 kb)

Additional file 3: **Figure S3.** Flow-sorting fresh frozen (FF) TNBC tissue samples. DNA content analysis of diploid and aneuploid populations flow-sorted from FF TNBC tissues. DNA content and cell cycle were analyzed by using the MultiCycle software program (Phoenix Flow Systems, San Diego, CA, USA). Abbreviation: *TNBC* triple-negative breast cancer. (PPTX 500 kb)

Additional file 4: **Figure S4.** FISH validation of high-level 9p24.1 amplicon. **A)** DNA content histogram of flow-sorted TNBC-11. **B)** Chromosome 9 Comparative Genomic Hybridization plot with high-level (log_2_ ratio >4) gain of JAK2 locus (arrow) at 9p24.1. **C)** Multi-color FISH assay [5′JAK2[9p24](green)/ 3′JAK2[9p24](red)/CEN 9(aqua)] image indicates more than 21 intact JAK2 signals and 1–3 CEN 9 signals. Abbreviations: *FISH* fluorescence *in situ* hybridization, *TNBC* triple-negative breast cancer. (PPTX 948 kb)

Additional file 5: **Figure S5.** FISH validation of 9p24.1 amplicon. **A)** DNA content histogram of flow-sorted TNBC-29. **B)** Chromosome 9 Comparative Genomic Hybridization plot with (log_2_ ratio >1) gain of JAK2 locus (arrow) at 9p24.1. **C)** Multi-color FISH assay [5′JAK2[9p24](green)/ 3′JAK2[9p24](red)/CEN 9(aqua)] image indicates 3–5 intact JAK2 signals and 2–3 CEN 9 signals. Abbreviations: *FISH* fluorescence *in situ* hybridization, *TNBC* triple-negative breast cancer. (PPTX 776 kb)

Additional file 6: **Figure S6.** FISH validation of 9p24.1 copy number loss. **A)** DNA content histogram of flow-sorted TNBC-8. **B)** Chromosome 9 Comparative Genomic Hybridization plot with (log_2_ ratio − 1) loss of JAK2 locus (arrow) at 9p24.1. **C)** Multi-color FISH assay [5′JAK2[9p24](green)/ 3′JAK2[9p24](red)/CEN 9(aqua)] image indicates 0–2 intact JAK2 signals and 1–4 CEN 9 signals. Abbreviations: *FISH* fluorescence *in situ* hybridization, *TNBC* triple-negative breast cancer. (PPTX 700 kb)

Additional file 7: **Figure S7.** TNBC with JAK1 homozygous deletion. **A)** DNA content histogram of flow-sorted TNBC-51. **B)** Whole genome CNV profile of 3.5 N aneuploid TNBC-51 genome. **C)** Homozygous deletion at 1p31.3 includes the JAK1 locus. Red shaded area denotes ADM2-defined CNV interval. Abbreviations: *CNV* copy number variant, *TNBC* triple-negative breast cancer. (PPTX 226 kb)

Additional file 8: **Figure S8.** TNBCs with high mutation loads and predicted neoepitopes. **A, D)** DNA content histogram of flow-sorted TNBC-11 and TNBC-12. **B–E)** Whole genome CNV profiles of flow-sorted tumors. **C–F)** IGV view of DCLRE1C and ALKBH5 somatic mutations. PD-L1 staining and location (*NT* non-tumor cells, *T/NT* tumor plus non-tumor cells) are presented for each case. Abbreviations: *CNV* copy number variant, *TNBC* triple-negative breast cancer. (PPTX 196 kb)
