# Supplementary material for: The association of genomic lesions and PD-1/PD-L1 expression in resected triple-negative breast cancers
Source: Breast Cancer Res. 2018 Jul 11;20:71. doi: 10.1186/s13058-018-1004-0 (PMC6042255; doi:10.1186/s13058-018-1004-0)
Supplement: Supplementary file 1 — Figure S1. Workflow and analyses of TNBC cohort. Fifty-five resections were screened for PD-1 and PD-L1 expression with IHC. Biopsies from 48 resections were flow-sorted and profiled for CNVs. Combined IHC and CNV data were obtained from 46 cases in this study. Abbreviations: CNV copy number variant, IHC immunohistochemistry, TNBC triple-negative breast cancer. (PPTX 74 kb) [file 13058_2018_1004_MOESM1_ESM.pptx]

## Slide 1
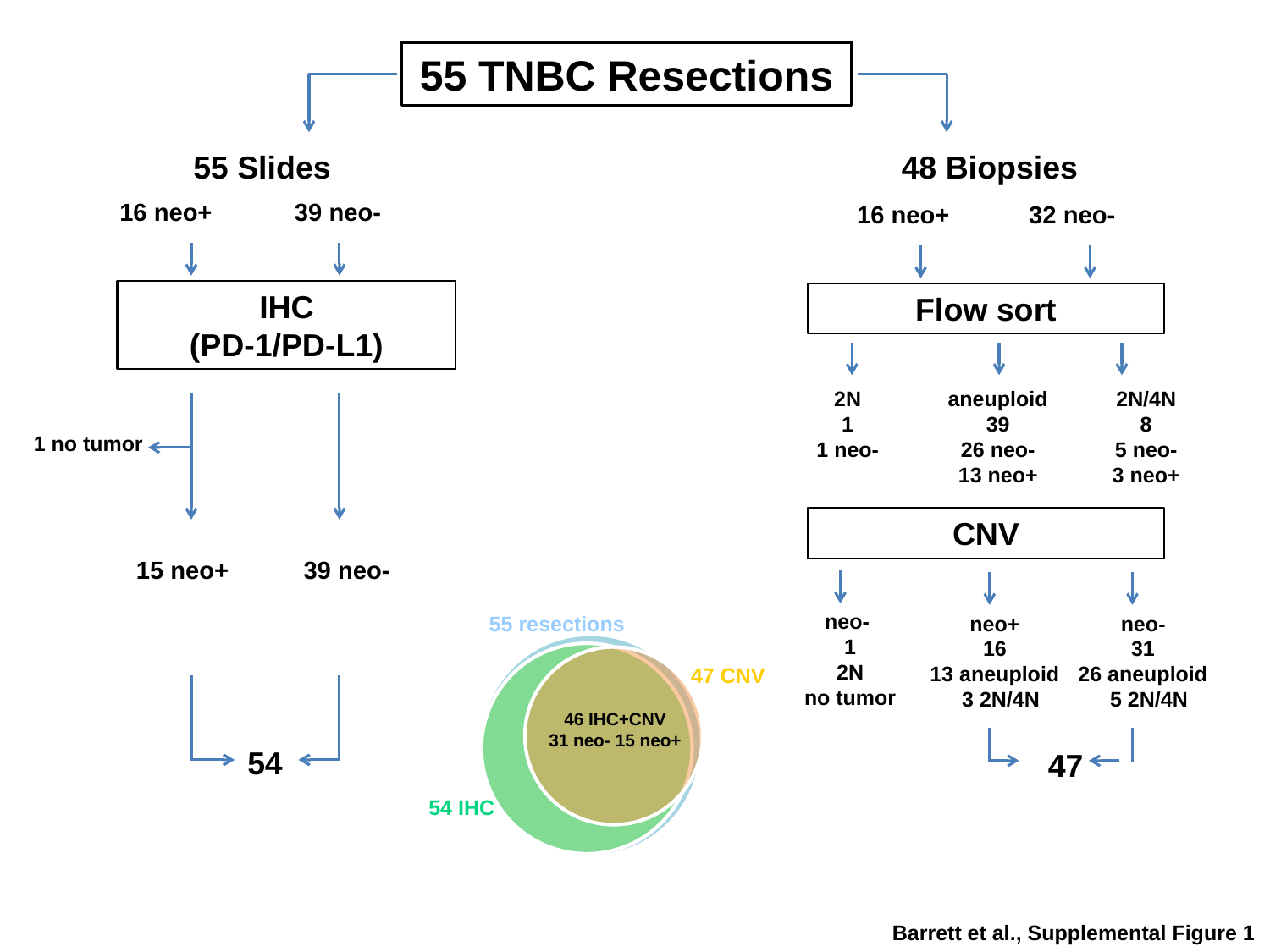

55 TNBC Resections
55 Slides
48 Biopsies
16 neo+
39 neo-
16 neo+
32 neo-
IHC
(PD-1/PD-L1)
Flow sort
2N
1
1 neo-
aneuploid
39
26 neo-
13 neo+
2N/4N
8
5 neo-
3 neo+
1 no tumor
CNV
15 neo+
39 neo-
neo-
1
2N
no tumor
55 resections
neo+
16
13 aneuploid
 3 2N/4N
neo-
31
26 aneuploid
 5 2N/4N
47 CNV
46 IHC+CNV
31 neo- 15 neo+
54
47
54 IHC
Barrett et al., Supplemental Figure 1
