# Supplementary figures and images for: The association of genomic lesions and PD-1/PD-L1 expression in resected triple-negative breast cancers
Source: Breast Cancer Res. 2018 Jul 11;20:71. doi: 10.1186/s13058-018-1004-0 (PMC6042255; doi:10.1186/s13058-018-1004-0)

## Slide 1
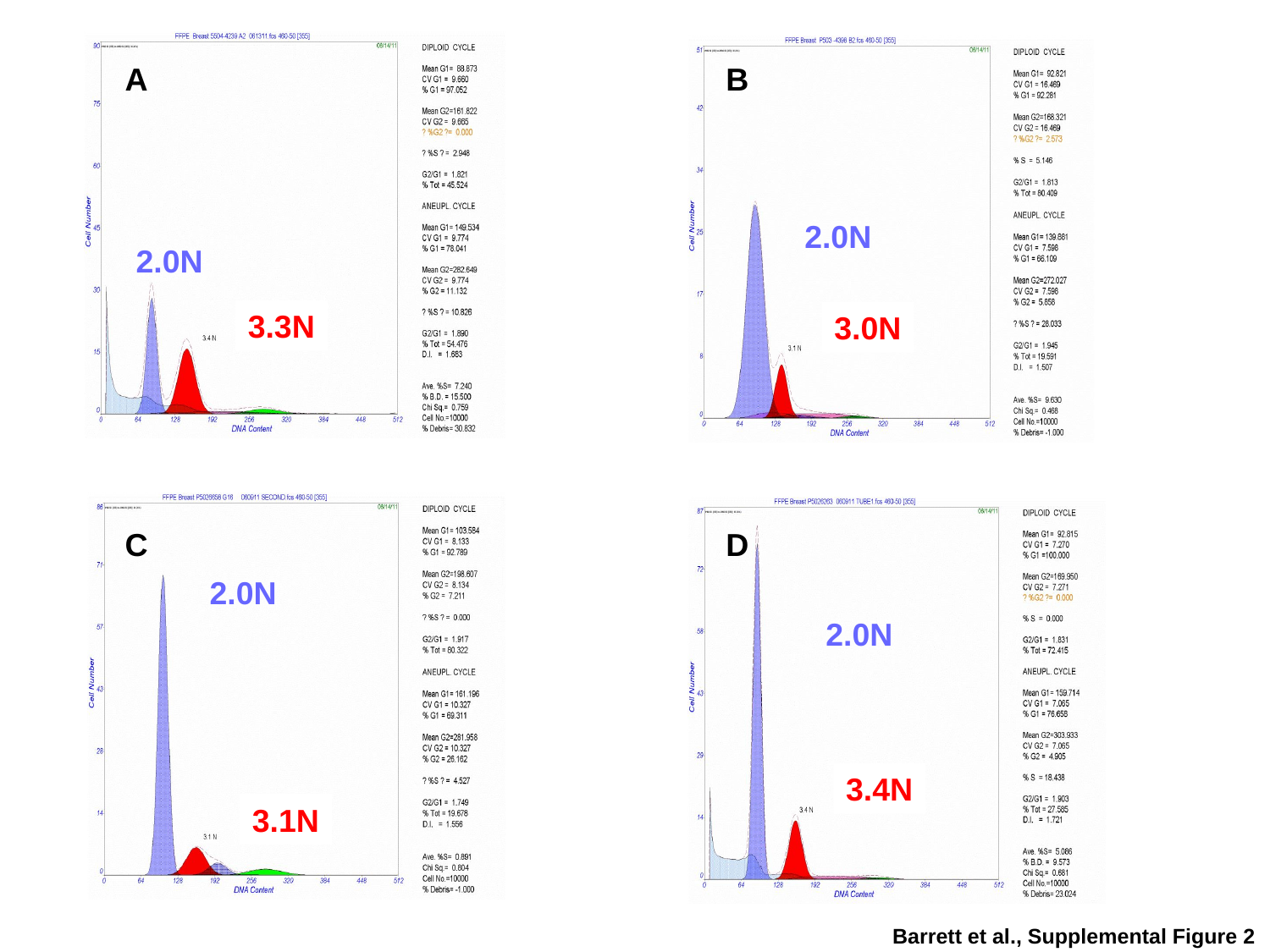

2.0N
3.3N
2.0N
3.0N
A
B
2.0N
3.1N
2.0N
3.4N
C
D
Barrett et al., Supplemental Figure 2

Supplement: Supplementary file 2 — Figure S2. Flow-sorting formalin-fixed paraffin-embedded (FFPE) TNBC tissue samples. DNA content analysis of diploid and aneuploid populations flow-sorted from FFPE TNBC tissues. DNA content and cell cycle were analyzed by using the MultiCycle software program (Phoenix Flow Systems, San Diego, CA, USA). Abbreviation: TNBC triple-negative breast cancer. (PPTX 603 kb) [file 13058_2018_1004_MOESM2_ESM.pptx]

## Slide 1
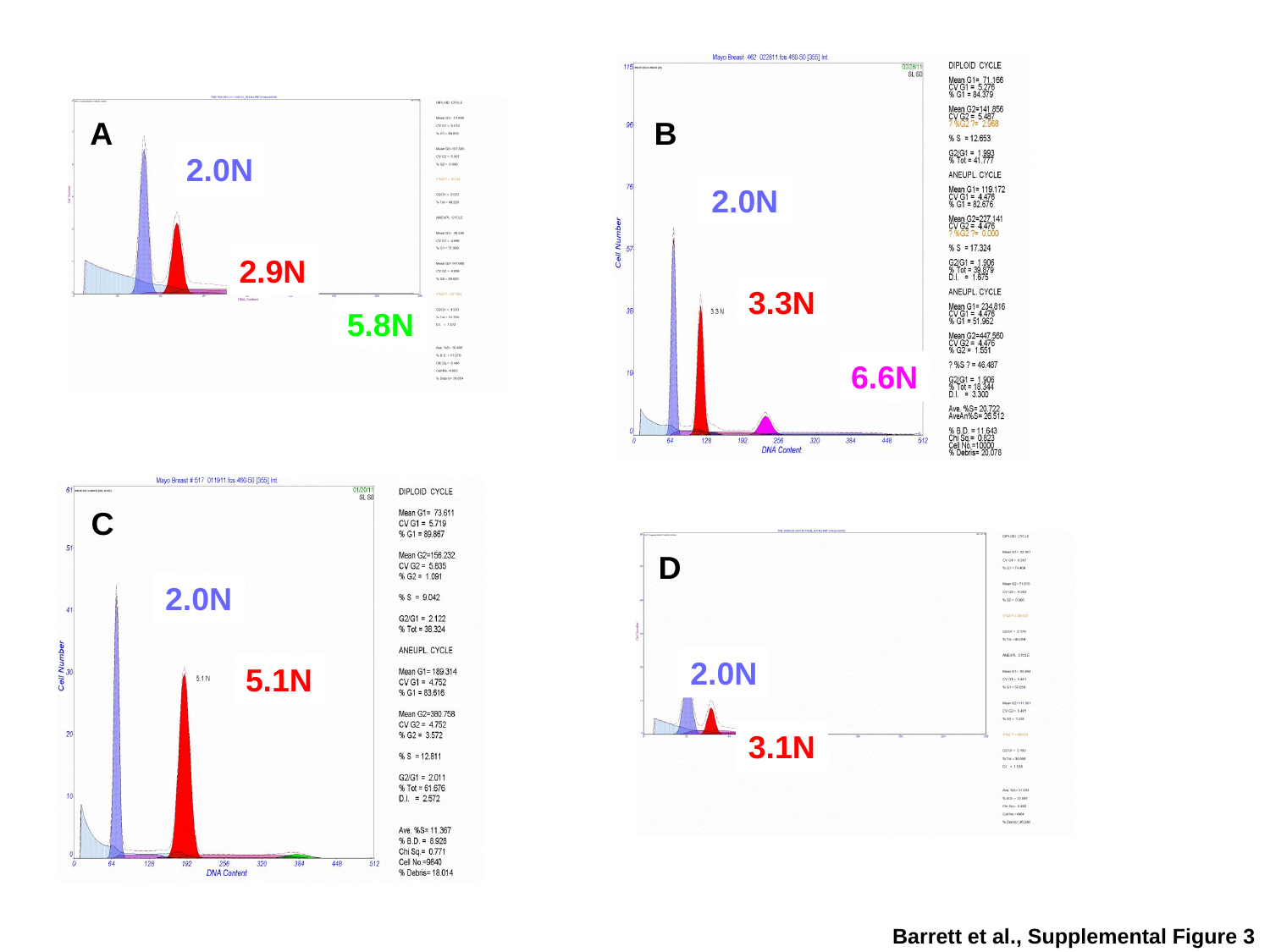

2.0N
3.3N
6.6N
2.0N
2.9N
5.8N
A
B
2.0N
5.1N
C
D
2.0N
3.1N
Barrett et al., Supplemental Figure 3

Supplement: Supplementary file 3 — Figure S3. Flow-sorting fresh frozen (FF) TNBC tissue samples. DNA content analysis of diploid and aneuploid populations flow-sorted from FF TNBC tissues. DNA content and cell cycle were analyzed by using the MultiCycle software program (Phoenix Flow Systems, San Diego, CA, USA). Abbreviation: TNBC triple-negative breast cancer. (PPTX 500 kb) [file 13058_2018_1004_MOESM3_ESM.pptx]

## Slide 1
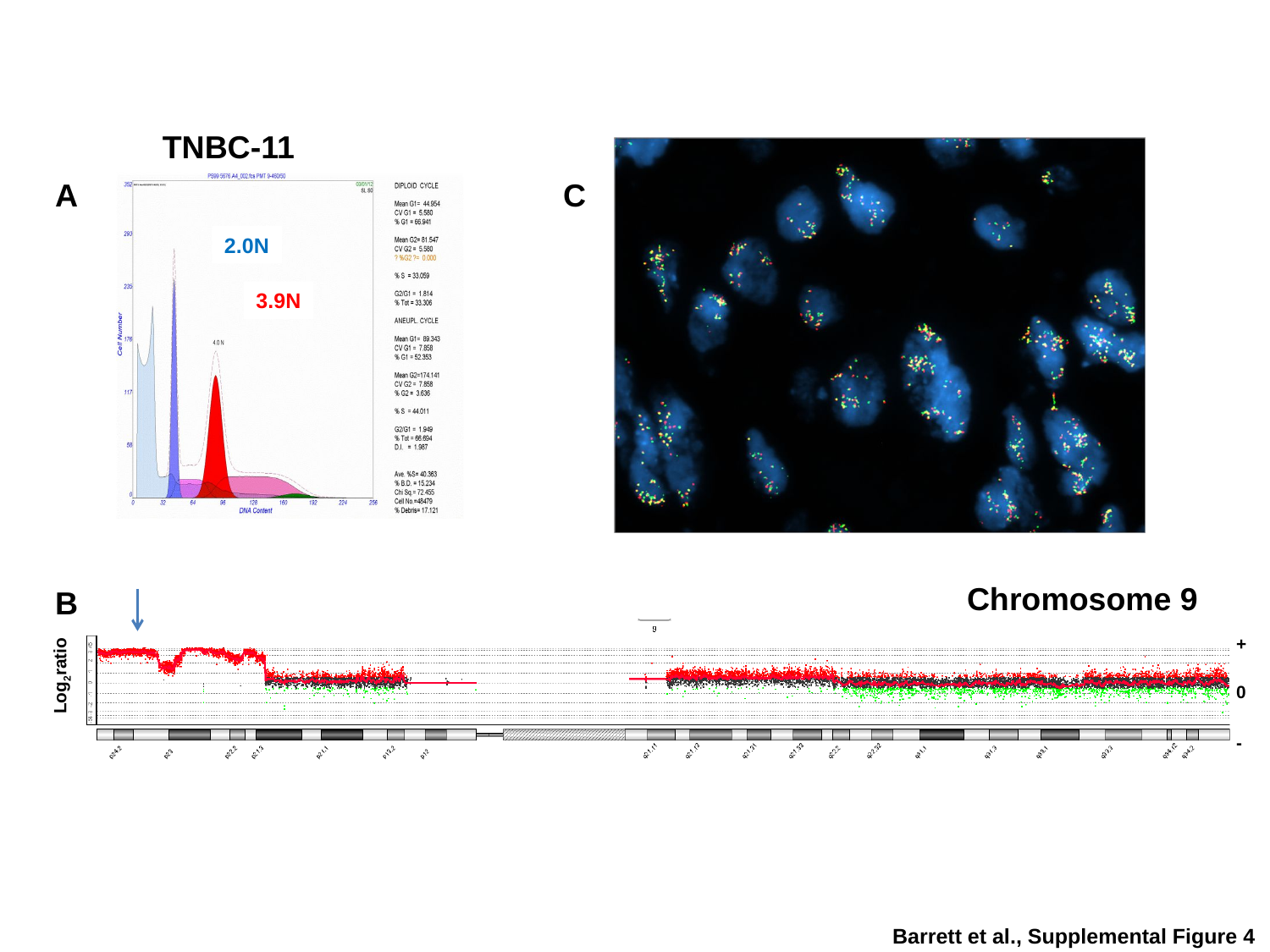

TNBC-11
A
C
2.0N
3.9N
Chromosome 9
B
+
0
-
Log2ratio
Barrett et al., Supplemental Figure 4

Supplement: Supplementary file 4 — Figure S4. FISH validation of high-level 9p24.1 amplicon. A) DNA content histogram of flow-sorted TNBC-11. B) Chromosome 9 Comparative Genomic Hybridization plot with high-level (log2 ratio >4) gain of JAK2 locus (arrow) at 9p24.1. C) Multi-color FISH assay [5′JAK2[9p24](green)/ 3′JAK2[9p24](red)/CEN 9(aqua)] image indicates more than 21 intact JAK2 signals and 1–3 CEN 9 signals. Abbreviations: FISH fluorescence in situ hybridization, TNBC triple-negative breast cancer. (PPTX 948 kb) [file 13058_2018_1004_MOESM4_ESM.pptx]

## Slide 1
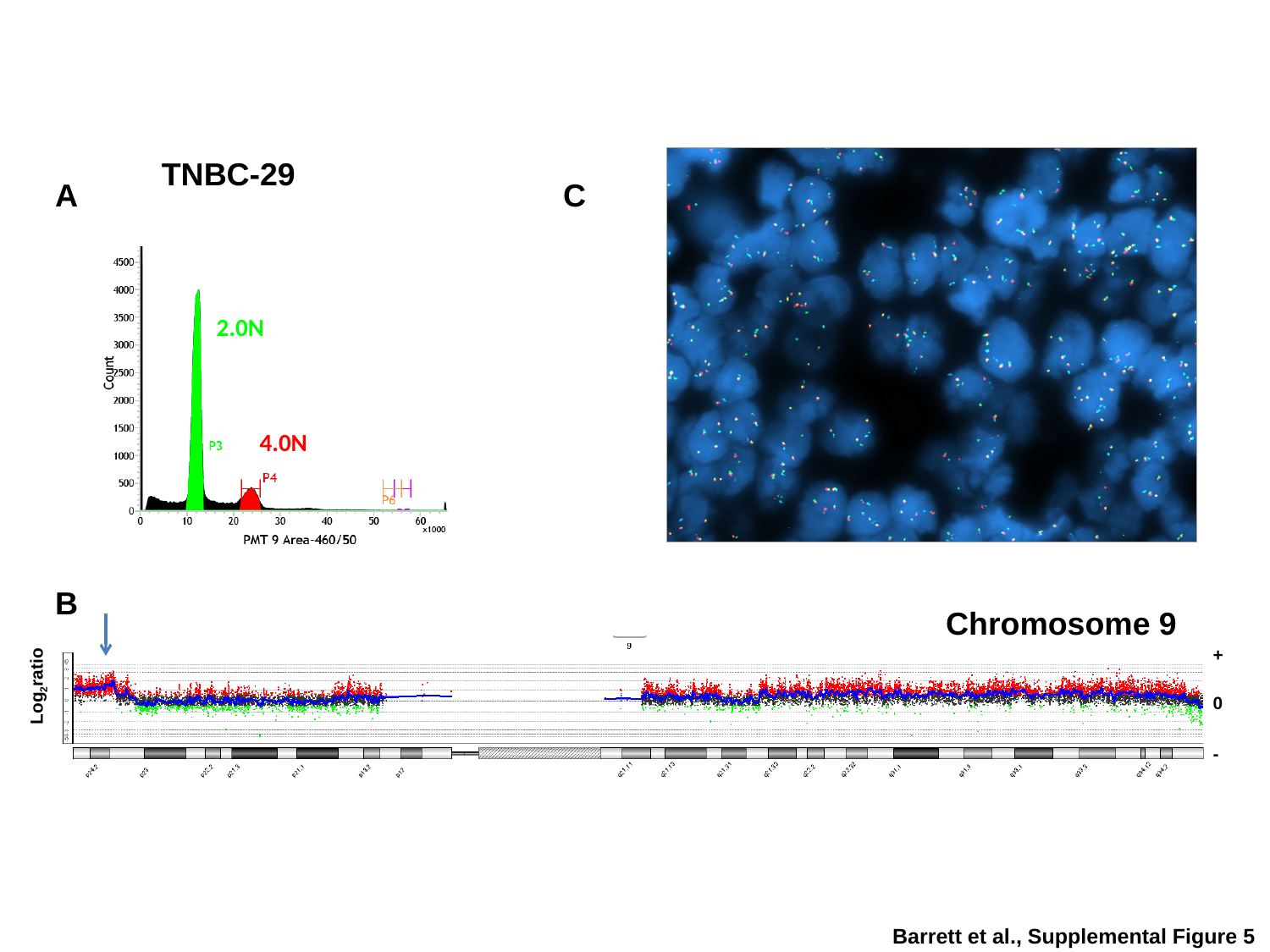

TNBC-29
A
C
2.0N
4.0N
B
Chromosome 9
+
0
-
Log2ratio
Barrett et al., Supplemental Figure 5

Supplement: Supplementary file 5 — Figure S5. FISH validation of 9p24.1 amplicon. A) DNA content histogram of flow-sorted TNBC-29. B) Chromosome 9 Comparative Genomic Hybridization plot with (log2 ratio >1) gain of JAK2 locus (arrow) at 9p24.1. C) Multi-color FISH assay [5′JAK2[9p24](green)/ 3′JAK2[9p24](red)/CEN 9(aqua)] image indicates 3–5 intact JAK2 signals and 2–3 CEN 9 signals. Abbreviations: FISH fluorescence in situ hybridization, TNBC triple-negative breast cancer. (PPTX 776 kb) [file 13058_2018_1004_MOESM5_ESM.pptx]

## Slide 1
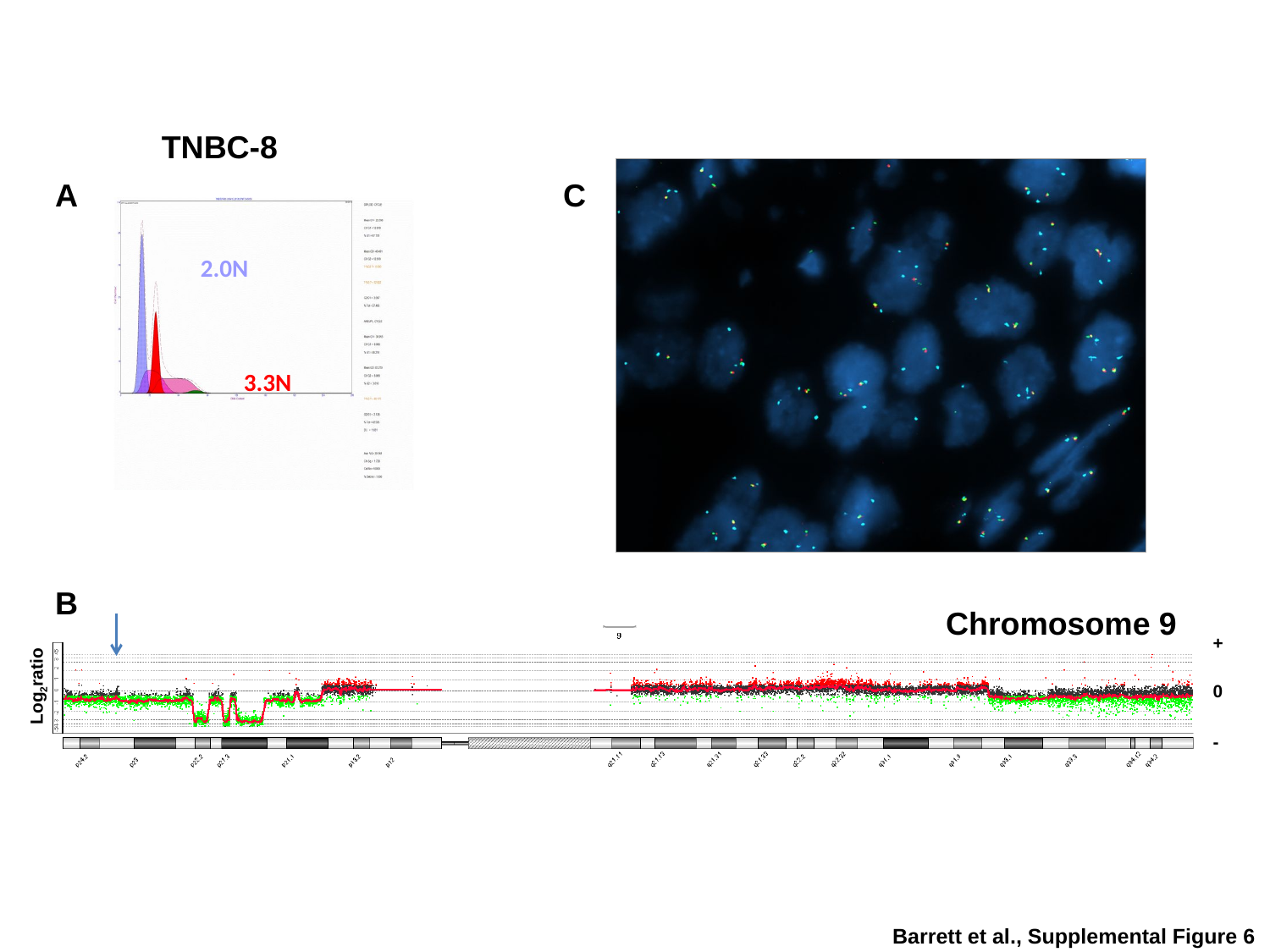

TNBC-8
A
C
2.0N
3.3N
B
Chromosome 9
+
0
-
Log2ratio
Barrett et al., Supplemental Figure 6

Supplement: Supplementary file 6 — Figure S6. FISH validation of 9p24.1 copy number loss. A) DNA content histogram of flow-sorted TNBC-8. B) Chromosome 9 Comparative Genomic Hybridization plot with (log2 ratio − 1) loss of JAK2 locus (arrow) at 9p24.1. C) Multi-color FISH assay [5′JAK2[9p24](green)/ 3′JAK2[9p24](red)/CEN 9(aqua)] image indicates 0–2 intact JAK2 signals and 1–4 CEN 9 signals. Abbreviations: FISH fluorescence in situ hybridization, TNBC triple-negative breast cancer. (PPTX 700 kb) [file 13058_2018_1004_MOESM6_ESM.pptx]
