# Supplementary material for: The association of genomic lesions and PD-1/PD-L1 expression in resected triple-negative breast cancers
Source: Breast Cancer Res. 2018 Jul 11;20:71. doi: 10.1186/s13058-018-1004-0 (PMC6042255; doi:10.1186/s13058-018-1004-0)
Supplement: Supplementary file 7 — Figure S7. TNBC with JAK1 homozygous deletion. A) DNA content histogram of flow-sorted TNBC-51. B) Whole genome CNV profile of 3.5 N aneuploid TNBC-51 genome. C) Homozygous deletion at 1p31.3 includes the JAK1 locus. Red shaded area denotes ADM2-defined CNV interval. Abbreviations: CNV copy number variant, TNBC triple-negative breast cancer. (PPTX 226 kb) [file 13058_2018_1004_MOESM7_ESM.pptx]

## Slide 1
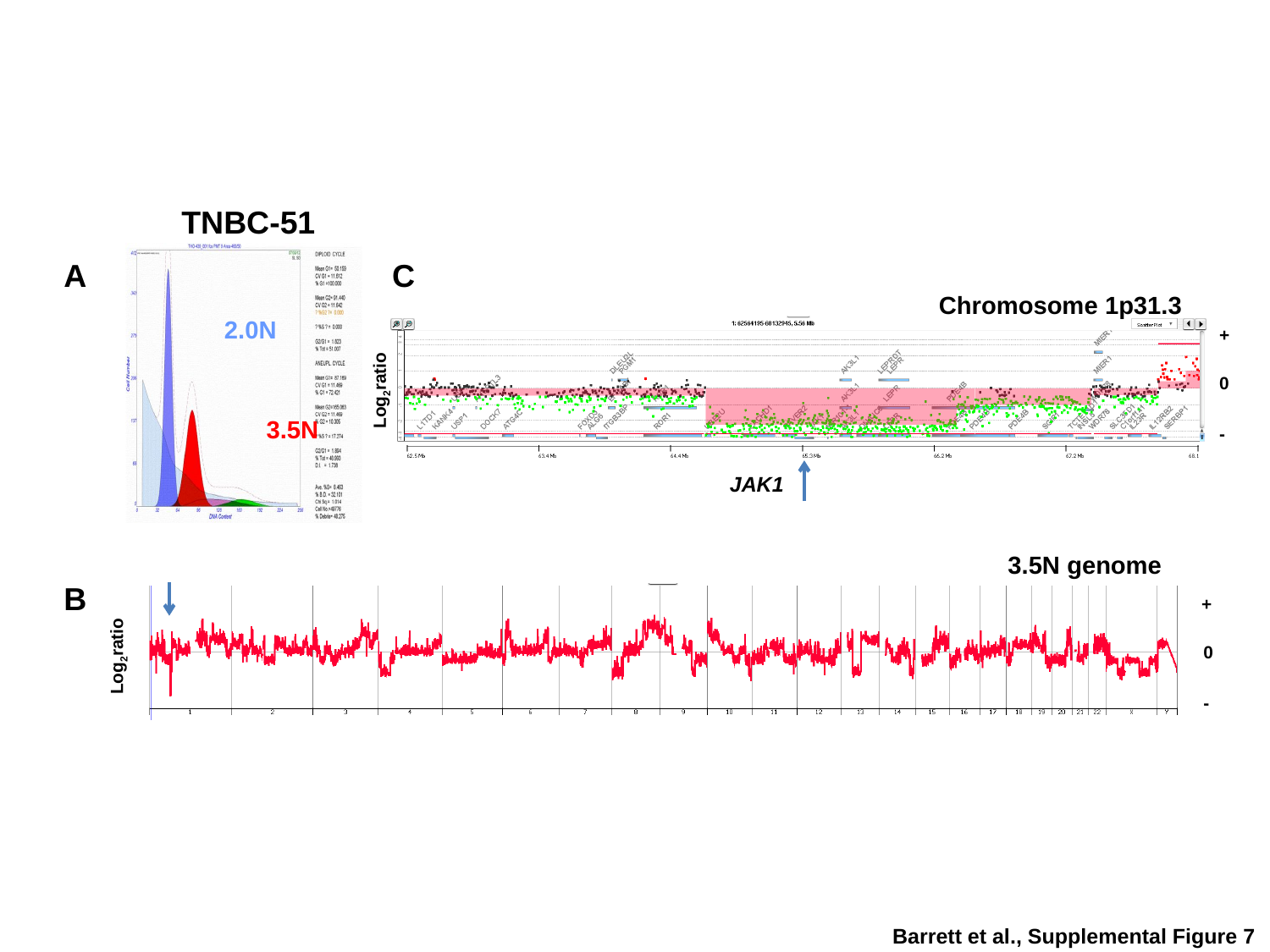

TNBC-51
A
C
Chromosome 1p31.3
2.0N
+
0
-
Log2ratio
3.5N
JAK1
3.5N genome
B
+
0
-
Log2ratio
Barrett et al., Supplemental Figure 7
