# Supplementary material for: The association of genomic lesions and PD-1/PD-L1 expression in resected triple-negative breast cancers
Source: Breast Cancer Res. 2018 Jul 11;20:71. doi: 10.1186/s13058-018-1004-0 (PMC6042255; doi:10.1186/s13058-018-1004-0)
Supplement: Supplementary file 8 — Figure S8. TNBCs with high mutation loads and predicted neoepitopes. A, D) DNA content histogram of flow-sorted TNBC-11 and TNBC-12. B–E) Whole genome CNV profiles of flow-sorted tumors. C–F) IGV view of DCLRE1C and ALKBH5 somatic mutations. PD-L1 staining and location (NT non-tumor cells, T/NT tumor plus non-tumor cells) are presented for each case. Abbreviations: CNV copy number variant, TNBC triple-negative breast cancer. (PPTX 196 kb) [file 13058_2018_1004_MOESM8_ESM.pptx]

## Slide 1
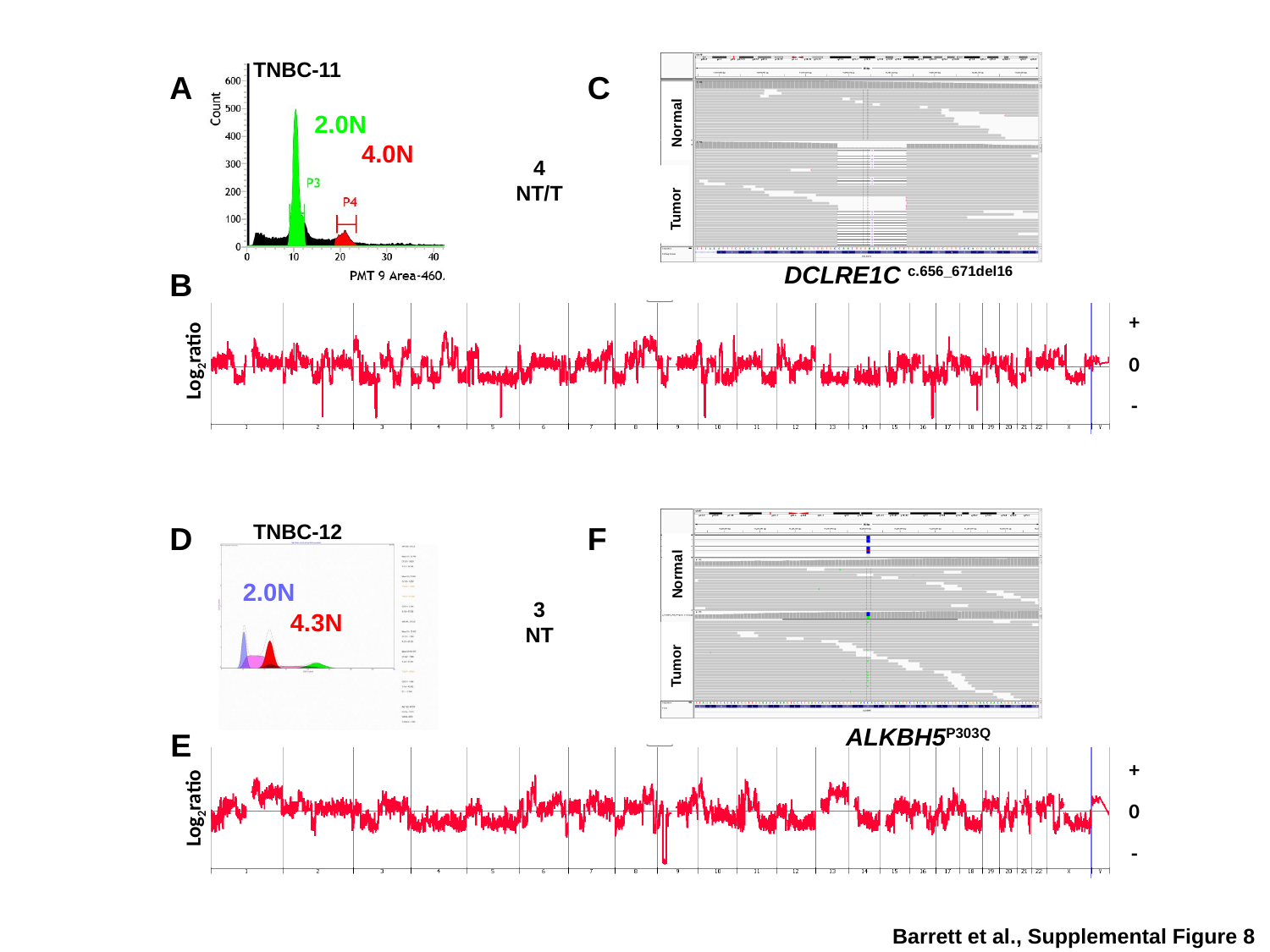

TNBC-11
Normal
Tumor
DCLRE1C c.656_671del16
C
A
2.0N
4.0N
4
NT/T
B
+
0
-
Log2ratio
Normal
Tumor
ALKBH5P303Q
D
TNBC-12
F
2.0N
4.3N
3
NT
E
+
0
-
Log2ratio
Barrett et al., Supplemental Figure 8
